# Contribution of oenocytes and pheromones to courtship behaviour in Drosophila

**DOI:** 10.1186/1471-2091-10-21

**Published:** 2009-08-11

**Authors:** Claude Wicker-Thomas, Ilhem Guenachi, Youssouf F Keita

**Affiliations:** 1 CNRS, UPR9034, LEGS and University Paris Sud, 91198 Gif sur Yvette Cedex, France; 2 NAMC, UMR 9620, University Paris Sud, 91405 Orsay Cedex, France

## Abstract

**Background:**

In Drosophila, cuticular sex pheromones are long-chain unsaturated hydrocarbons synthesized from fatty acid precursors in epidermal cells called oenocytes. The species *D. melanogaster *shows sex pheromone dimorphism, with high levels of monoenes in males, and of dienes in females. Some biosynthesis enzymes are expressed both in fat body and oenocytes, rendering it difficult to estimate the exact role of oenocytes and of the transport of fatty acids from fat body to oenocytes in pheromone elaboration. To address this question, we RNAi silenced two main genes of the biosynthesis pathway, *desat1 *and *desatF*, in the oenocytes of *D. melanogaster*, without modifying their fat body expression.

**Results:**

Inactivation of *desat1 *in oenocytes resulted in a 96% and 78% decrease in unsaturated hydrocarbons in males and females, respectively. Female pheromones (dienes) showed a decrease of 90%. Inactivation of *desatF*, which is female-specific and responsible for diene formation, resulted in a dramatic loss of pheromones (-98%) paralleled with a two-fold increase in monoenes. Courtship parameters (especially courtship latency) from wild-type males were more affected by *desat1 *knocked-down females (courtship latency increased by four fold) than by *desatF *knocked-down ones (+65% of courtship latency).

The number of transcripts in oenocytes was estimated at 0.32 and 0.49 attomole/μg for *desat1 *in males and females, respectively, about half of the total transcripts in a fly. There were only 0.06 attomole/μg *desatF *transcripts in females, all located in the oenocytes.

**Conclusion:**

Knock-down results for *desat1 *suggest that there must be very little transport of unsaturated precursors from fat body to the oenocytes, so pheromone synthesis occurs almost entirely through the action of biosynthesis enzymes within the oenocytes. Courtship experiments allow us to discuss the behavioral role of diene pheromones, which, under special conditions, could be replaced by monoenes in *D. melanogaster*. A possible explanation is given of how pheromones could have evolved in species such as *D. simulans*, which only synthesize monoenes.

## Background

In insects, sex pheromones, together with visual and acoustic clues, play a large role in the courtship behaviour preceding mating [1-4]. In Drosophila, pheromones are long-chain hydrocarbons which appear to be synthesized in large epidermal cells called oenocytes and deposited on the cuticle [5]. They have been widely studied and their biosynthesis is partly known: fatty acid precursors are desaturated by acyl-CoA-desaturases and elongated to give very long chain fatty acids, which are eventually decarboxylated to give hydrocarbons [6,7]. Among the *Drosophila melanogaster *hydrocarbons, only unsaturated ones have been shown to have a behavioral role: principally monoenes, unsaturated in position 7 in males and dienes unsaturated in positions 7 and 11 in females. The main pheromones are 7-tricosene (7-T; 23:1) and 7-pentacosene (7-P; 25:1) in males, and 7,11-heptacosadiene (7,11-HD; 27:2) and 7,11-nonacosadiene (7,11-ND; 29:2) in females. Some other hydrocarbons with one unsaturation, 7-pentacosene and 7-heptacosene (7-H; 27:1), can also elicit – at a lower level – courtship behavior [1,8].

In the biosynthesis of pheromones, two desaturases can perform unsaturations: Desat1 enzyme catalyzes the conversion of palmitic and stearic acids into palmitoleic and oleic acids, which serve as substrates for the synthesis of various lipids and also of 7-unsaturated hydrocarbons [9,10]. *desat1 *gene is expressed in both males and females in a number of tissues, including fat body, which is the main site of production of fatty acids, and oenocytes, which is the site of production of hydrocarbons and is involved in hydrocarbon metabolism [11]. Female fly mutants for *desat1 *have fewer pheromones and are less attractive to wild-type males, leading to increased courtship latency [12-14]. In *desat1 *mutants, lipid metabolism is also severely impaired, and characterized by a dramatic decrease in both saturated and unsaturated fatty acid production [14]. The second enzyme is DesatF, which introduces a second double bond in the fatty acid precursors. The gene, only expressed in females, seems specifically expressed in oenocytes; a line knocked-down for *desatF *has been generated in the laboratory. The expression of *desatF RNAi *in fat body alone had no effect; the same expression induced in both oenocytes and fat body led to a large decrease in female pheromone production and also to less courtship from wild-type males [15].

As *desat1 *pleiotropic effects could be due to its expression in different tissues, we wanted to dissociate the effects of *desat1 *in oenocytes from those in fat body. We wondered whether oenocytes could – alone – account for the synthesis of unsaturated hydrocarbons. Therefore, we used one GAL4 driver targeting expression in the oenocytes without affecting the fat body. Overexpression of *desat1 *in oenocytes resulted in a small increase in unsaturated hydrocarbons. With this same driver, flies knocked-down for *desat1 *showed a dramatic loss of all the unsaturated hydrocarbons. We quantified *desat1 *transcripts in the oenocytes by the comparison of the numbers of transcripts in flies expressing or knocked-down for *desat1 *in oenocytes. We also studied male courtship behavior toward the *desat1 *knocked-down females. We compared these effects with the effects of *desatF RNAi *expressed in the oenocytes. *desatF *knock-down in females led to a large increase in monoenes at the expense of dienes, which were almost completely eliminated. We studied the effect of these *desatF *knocked-down females on wild-male courtship behavior and quantified the number of *desatF *transcripts. Results show that wild-type male behavior toward *desat1 *and *desatF RNAi *females was very different. The role of unsaturated hydrocarbons on male courtship behavior is discussed.

## Results

### Effect of *desat1 *overexpression on hydrocarbons

**Figure 1:**
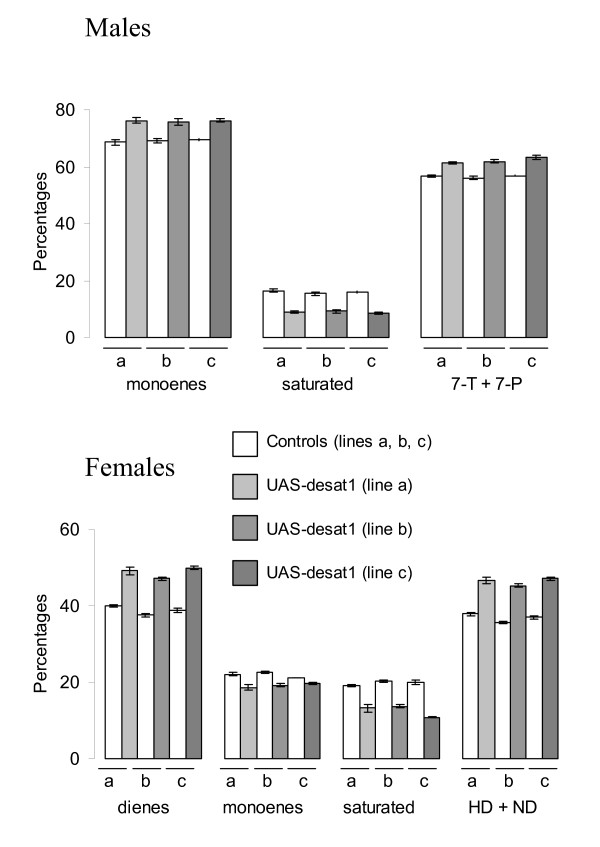
**Overexpression of *desat1 *in oenocytes results in an increase of unsaturated hydrocarbons (monoenes and dienes) and a decrease in saturated ones**. Mean hydrocarbons (± SEM) of 4-day-old male and female flies of wild-type (white) or expressing *desat1 *(grey) under 1407-GAL4 driver. Three different *UAS-desat1 *lines (a, b, c) were used. n = 10 for all tests. All the hydrocarbon differences between control (1407-GAL4/+) and *desat1 *overexpressing lines (1407-GAL4/*UAS-desat1*) were significantly different (*P *< 0.05, *Student's t*-test).

Hydrocarbons from flies overexpressing desat1 under the control of *1407-GAL4 *resulted in a small but significant increase in unsaturated hydrocarbons (+ 10 to 16%), at the expense of saturated hydrocarbons (-15 to 46%). In females, total diene and pheromone (HD+ND) levels were particularly enhanced (+ 23 to 28%); in males, the pheromone level (7-T + 7-P) was increased from 8 to 12%, depending on the line (Figure 1).

### Effect of *desat1 *knock-down on hydrocarbons (Figure 2)

**Figure 2:**
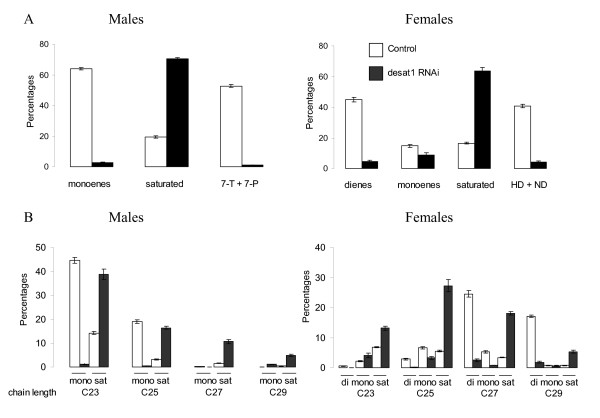
**RNAi knock-down of *desat1 *in oenocytes leads to a dramatic decrease in unsaturated hydrocarbons counter-balanced by an increase in saturated hydrocarbons**. A. Percentages of different classes of hydrocarbons (dienes, monoenes, saturated, pheromones) in control (white) and RNAi (black) flies. B. Percentages of the specific hydrocarbons according to their class and their length. All the hydrocarbon differences between control (1407-GAL4/+) and *desat1 *knocked-down lines (1407-GAL4/*UAS-desat1 RNAi*) were significantly different (*P *< 0.001, *Student's t*-test).

When *1407-GAL4 *driver was used to target *desat1RNAi*, no lethality occurred and flies seemed unaffected in their behavior (locomotor activity). However, their hydrocarbon profiles were dramatically affected, with an almost complete disappearance of unsaturated hydrocarbons (-96% and -78% in males and females, respectively). The level of saturated linear hydrocarbons was multiplied by 4 in both sexes. In females, total dienes (and pheromones) were decreased by 90%, monoenes by 40%.

### Effect of *desatF *knock-down on hydrocarbons (Figure 3)

**Figure 3:**
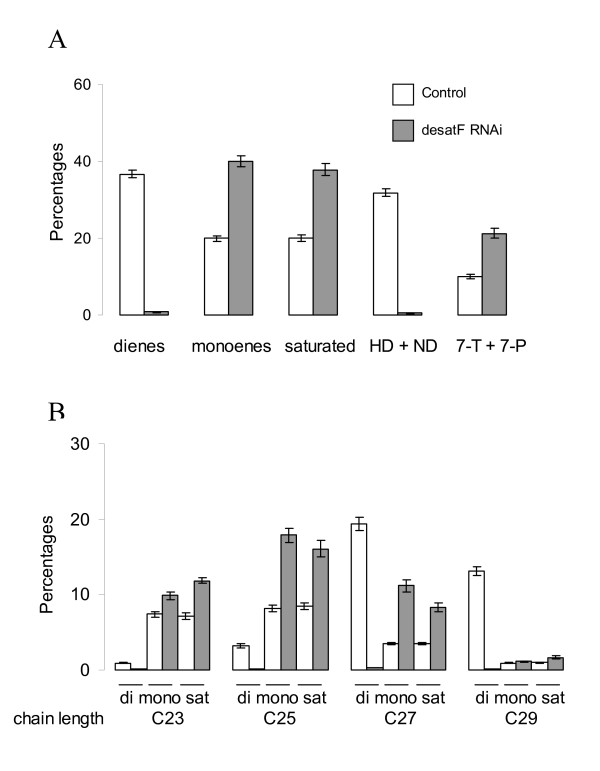
**RNAi knock-down of *desatF *in oenocytes leads to a dramatic decrease in dienes counter-balanced by an increase in monoenes**. A. Percentages of different classes of hydrocarbons (dienes, monoenes, saturated, female pheromones, male-type pheromones) B. Percentages of the specific hydrocarbons according to their class and their length. All the hydrocarbon differences between control (1407-GAL4/+) and *desatF *knocked-down lines (1407-GAL4/*UAS-desatF RNAi*) were significantly different (*P *< 0.001, *Student's t*-test).

The *RNAi *silencing of *desatF *expression in females under *1407-GAL4 *driver resulted in an increase (+ 27%) in linear saturated hydrocarbons; the amount of dienes and pheromones was decreased by 98%, that of monoenes was doubled.

### Courtship behavior

**Figure 4:**
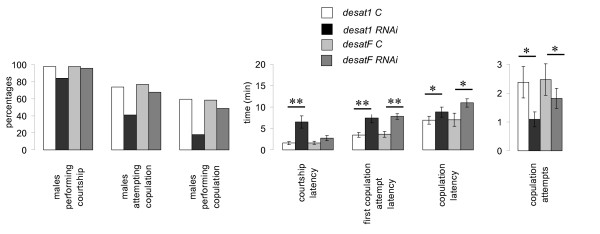
**RNAi knock-down of *desat1 *and *desatF *in females had very different effects on wild-type male courtship, with more marked effects in the former females**. Mean courtship parameters (± SEM) of 4-day-old tester Canton-S male flies with *desat1 RNAi *females (controls in white, *desat1 RNAi *in black) and *desatF RNAi *females (controls in light grey, *desatF RNAi *in grey). n > 40 for all tests. Means with * and ** were significantly different with the Mann-Whitney *U*-test (*P *< 0.05 and 0.01, respectively).

The percentages of wild-type males performing courtship toward the two types of females (control and *desat1RNAi*) were similar, but courtship latency was 4 times higher when the female was *desat1RNAi *(Figure 4). Males attempting copulation and males succeeding in copulation were fewer (-45% and -70%, respectively) and times required for these behaviours were increased (+ 112% and + 28%, respectively). The total copulation attempts were also decreased (-54%) when males were in the presence of *desat1RNAi *females.

The courtship parameters were less affected when males were put with *desatFRNAi *females: there were only 12% fewer copulation attempts and 15% less copulation, respectively. Courtship, the first copulation attempt and copulation latencies were increased by 65%, 115% and 55%, respectively.

### Quantification of transcripts

The number of *desat1 *transcripts was 1.6 times higher in females than in males (Table 1). After RNAi expression under 1407-GAL4, the number of transcripts was halved in both sexes. The number of *desat1 *transcripts in GAL4-expressing cells was therefore estimated at 0.32 and 0.49 attomoles/μg total RNA in males and females, respectively.

**Table 1:** About half of the total *desat1 *transcripts are located in the oenocytes.

Genotype	transcripts	males	females
1407-GAL4/+	*desat1*	0.62 ± 0.11	1.02 ± 0.16
1407-GAL4/desat1 RNAi	*desat1*	0.32 ± 0.08	0.55 ± 0.03
1407-GAL4/+	*desatF*	0	0.06 ± 0.01

The number of ***desatF ***transcripts represents only 10% of the number of *desat1 *transcripts; all desatF transcripts are located in the female oenocytes.

The values represent the means of 4 determinations ± SEM of samples from separate experiments and are given in attomoles/μg total RNA.

The *desatF *transcripts could be detected only in 1407-GAL4/+ females, and their number was evaluated at 0.06 ± 0.01 attomoles/μg total RNA in 1407-GAL4/+ females.

## Discussion

Results show that knock-down of desaturase genes in oenocytes is sufficient to suppress or at least largely inhibit pheromone biosynthesis. The effect is particularly clear in males, where *desat1 *knock-down under 1407-GAL4 led to a 96% decrease in monoenic hydrocarbons. In females, the decrease is less dramatic (78%). The biosynthesis of the saturated hydrocarbons was enhanced, as a consequence of the lack of desaturation of fatty acid precursors. Decreased levels of unsaturated hydrocarbons and increased levels of saturated ones have previously been described in *P-desat1 *mutant and resulting excision lines [12,14]. However, the effects on hydrocarbons were relatively smaller, ranging from a 0 to about a 66% decrease in unsaturated hydrocarbons, depending on the lines. The fact that we never obtained an almost complete loss of hydrocarbons with *desat1 *mutants in former studies may be due to the pleiotropic nature of the mutation, which affects both fat body and hydrocarbons and also to the hypomorphic state of the mutant *desat1 *alleles [13]. The strongest mutant phenotypes (with a three-fold decrease in male monoenes) were leaner and poorly viable (many of the mutant flies died at the larval stage), with a two-fold loss of fat in the adults [14].

By contrast, *desat1 *knocked-down flies under 1407-GAL4 driver were perfectly viable and showed no difference from the control ones (apart from their hydrocarbon profile). The present results obtained with *desat1 *RNAi expressed mainly in the oenocytes imply that most of the biosynthesis of pheromones must be made *de novo *in the oenocytes. A transport of fatty acids and hydrocarbons by a transporter, the lipophorin, has been described, and radio-labelled fatty acid applied topically can serve as precursors for hydrocarbon synthesis [16]. Lipophorin is essential for selective lipid transport and energy production [17]. It also delivers hydrocarbons and sex pheromones from the oenocytes to the cuticular surface and could also play an important role in delivering hydrocarbons to specific tissues such as ovaries, the digestive tract and fat body [18-20]. The transport of fatty acids to the oenocytes does occur but seems relatively low: it has been estimated at about 2–4%, by comparing radioactivity found in crude extracts 3 or 6 days after topical application to that found in hydrocarbons [21] and may account for the low levels of pheromone biosynthesis remaining in *desat1 RNAi *flies.

For *desatF RNAi *flies, similar results have been described with another driver that targets expression in both oenocytes and fat body: the disappearance of dienes, which are replaced by monoenes of lower molecular weight [22]. This phenotype is due to the action of EloF, a female-specific elongase, which has been shown to elongate fatty acids to very long fatty acids (up to C30) [22]. This elongase is highly active in elongating dienic fatty acid precursors, and can also elongate monoenes, but with less efficiency. This would explain the particular profile of *desatF *RNAi females, very similar to that of males, with high levels of 7-T and 7-P, but with some other monoenes of a higher size, such as 7-heptacosene (C27:1). The main interest of the present study is to confirm that *desatF *expression is restricted to the oenocytes, and to compare the courtship of wild-type males toward both RNAi knocked-down females in the same conditions.

Overexpression of *desat1 *in oenocytes led to about a 10% and 25% increase in pheromones in males and females, respectively. The use of three independent lines gave similar results. These figures are relatively small, compared to the 57% increase in female pheromones resulting from the overexpression of *desatF *[15]. This moderate effect of *desat1 *overexpression could be due to a large expression of *desat1 *in oenocytes, which is already sufficient for a whole synthesis of hydrocarbons. Approximately half of the *desat1 *transcripts occur in the oenocytes. This figure is high, even if it may be overestimated, due to a faint expression of 1407-GAL4 in tissues such as the gut, besides a large expression in oenocytes [5]. It must also be remembered that *desat1 *is a pleiotropic gene, and its function in oenocytes is not limited to the synthesis of hydrocarbons, but also of other lipids, of fatty acids of the membranes, etc. On the other hand, *desatF*, which is specifically expressed in oenocytes, seems to be used only for pheromone production, as it is not expressed in species which have no dienic hydrocarbons [15]. The number of *desatF *transcripts is only 11% of the number of *desat1 *transcripts in oenocytes, and might be the limiting factor to a full synthesis of dienes. In fact, all the studies on the control of *Drosophila *female pheromones have shown an action of hormones (ecdysone) or neurotransmitters (dopamine) on the second desaturation step [23-25]. *desatF*, unlike *desat1*, seems very sensitive to hormonal conditions and appears to be the gene in which the regulation of female pheromones is exerted.

Another desaturase gene, *desat2*, has been evidenced in some African strain females that produce 5,9-HD in place of 7,11-HD [10]. This gene is responsible for the unsaturation of fatty acid precursors in position 5 and resembles the *desatF *gene, in that it seems to be used only for the production of 5,9-HD pheromones. A recent quantification of *desat2 *transcripts in these 5,9-HD females estimated a similar number of transcripts as for *desatF *(0.7 attomole/female) [11].

The role of unsaturated hydrocarbons on courtship behavior in *D. melanogaster *has been widely studied. The involvement of unsaturated hydrocarbons with 27 ± 2 carbons has been described [1,8], and the threshold action of the 7,11-HD has been evaluated as near the nanogram level [26]. In a previous study with a *desat1 *mutant, the four-fold decrease in both 7-monoenes and 7,11-dienes in females was accompanied by a 50% increase in courtship latency [14]. In the present study the effect of *desat1 *knock-down is followed by a 78% decrease in 7-monoenes (similar to that for *desat1 *mutant), but also a 90% decrease in 7,11-dienes and a four-fold increase in courtship latency. The longer delay to courtship for *desat1 *RNAi females, compared to the mutant ones, can thus be inferred from the lack of 7,11-dienes.

Knock-down of *desatF *gene in flies resulted in the disappearance of *desatF *transcripts, while only 2% of pheromones remained, and the levels of 7-P and 7-T were multiplied by two and three, respectively. These flies elicited the same courtship as wild-type females. Thus, monoenic hydrocarbons, such as 7-P and 7-H, can replace 7,11-dienes for courtship latency. However some parameters, such as copulation attempts and copulation latency, were much affected. In a previous study, similar results were obtained, using a more ubiquitous driver, with an effect only on copulation attempts and copulation latency [15]. These results tend to show that these two latter courtship parameters are affected by the presence of dienes.

*desat1 *is a gene which is essential for the fly, contrary to *desatF*. When *desatF *is knocked-down, larger amounts of monoenes are synthesized. Some of these monoenes, especially 7-heptacosene, have been shown to play a role in courtship [8,26]. When *desat1 *is knocked-down, only saturated hydrocarbons can be synthesized and none of these hydrocarbons have been shown to play a role in courtship. Actually, *desat1 *has a larger influence on pheromones and courtship, but, in wild-type flies, its expression level does not seem to vary much (wild-type flies have similar amounts of unsaturated hydrocarbons); at the opposite, wild-type flies exhibit different amounts of pheromones (dienes) and desatF seems very sensitive to environmental and hormonal changes (C W-T, non published data).

## Conclusion

*D. melanogaster *and *D. simulans *diverged only 2.5 million years ago [27]. Both species differ by their cuticular hydrocarbons, the second species being sexually monomorphic, with large amounts of 7-T and 7-P in males and females [28]. Both species also share the same ecological niche [29]. The gene *desatF *is present in *D. simulans *genome, but not transcribed [15]. In a previous paper, the rapid evolution of *desatF *promoter has been evidenced and the importance of *desatF *in speciation suggested [30]. The present study shows that a knock-out of *desatF *in *D. melanogaster *has dramatic effects on 7,11-dienes, but that these effects may be partially counter-balanced by the large increases in 7-monoenes at the courtship-behavior level. One might suppose that evolution first inactivated *desatF *in *D. simulans *ancestors, permitting – almost – normal courtship behavior and reproduction, followed by other modifications at the reception level. These studies should bring about better understanding of how speciation has occurred in the Drosophila subgroup.

## Methods

### Drosophila strains

Flies were maintained at 25°C with 12:12 light-dark (LD) cycles, on standard yeast/cornmeal/agar medium. Virgin females were collected 0–6 hr after emergence, grouped in food vials and used 4 days later for hydrocarbon, fatty acid analyses and courtship study. Males were also collected 0–6 hr after emergence and grouped in food vials for 4 days for hydrocarbon and fatty acid analyses. For courtship experiments, males from the Canton-S strain were isolated at emergence and kept individually in a food vial for 4 days.

One GAL4 line was used: 1407-GAL4 line targets expression in oenocytes and not in fat body [5].

The *UAS-desat1RNAi *line, referred to as desat1i, was provided by the Kyoto National Institute of Genetics . The *UAS-desatFRNAi *line, referred to as desatFi, was constructed and described previously [15].

### Construction of *UAS-desat1 *lines

The *desat1 *cDNA clone from Canton-S [7]. was cloned between the 5' *EcoRI *and 3' *XbaI *sites in *p*{*UAST*} plasmid [31]. Transgenic flies were generated by *P*-mediated germline transformation by Ann Mari Voie (EMBL) [32]. Multiple *UAS-desat1i *lines were obtained. Three lines, containing a single insert on the second chromosome, were used for genetic analysis.

### Crosses and data analysis

The *GAL4 *females were crossed to *UAS-desat1/Cy*, *UAS-desat1i/Cy *or *UAS-desatFi/Cy *males. The progeny was used for the experiments: the non Cy flies (expressing both GAL4 and the various UAS) were compared to the Cy control flies (expressing only GAL4). Data were analyzed using Student's *t*-test or one-way ANOVA.

### Hydrocarbon analysis

Hydrocarbons were extracted from individual flies in heptane and analyzed by gas chromatography (Perichrom) as previously described [10]. They were quantified as percentages of total hydrocarbons. Data are presented as mean percentages ± SEM of total hydrocarbons (n = 10 for all tests). Only the hydrocarbons that show variations are represented.

### Analysis of courtship behavior

One four-day-old virgin female (control or mutant) was tested with one four-day-old Canton-S male at 25°C for 20 min, as previously described [33]. The following courtship parameters were measured: the percentage of males performing courtship (wing vibration), the percentage of males attempting copulation, the percentage of males successfully copulating, courtship latency (time from the introduction of the male into the female-containing observation chamber until wing vibration occurs), copulation attempts latency, copulation latency, (n > 40 for all tests).

### Quantification of *desat1 *and *desatF *transcripts

The quantification of desaturase RNA was performed using the competitive quantitative polymerase chain reaction (PCR). This technique, which presents the advantage of high sensitivity, has been developed and has already been used for *desat1 *quantification [14]. In brief, a homologous internal DNA standard is used, differing only from *desat1 *cDNA by the presence of a short intron. The amplification of the two templates (*desat1 *cDNA together with the standard at different concentrations) permits *desat1 *transcripts to be quantified quite easily.

The same technique was developed for *desatF*. For the construction of the competitor, we used the two restriction sites *EcoRI *and *BamHI*, naturally occurring in the sequence and 465 pb distant from each other. *desatF *ORF (1068 bp) was cloned in a SK Bluescript, using *XbaI *and *HindIII *restriction sites of the plasmid. The clone was then digested with *EcoRI *and *BamHI*, liberating a 465 bp fragment and relegated, yielding to a 603 bp truncated orf, which was used as a competitor.

## Authors' contributions

CW-T did the pheromone analyses and wrote the paper; IG performed the *desat1 *and *desatF *quantification and participated to the writing; YFK performed the courtship behaviour and participated to the writing.
